# HuskinDB, a database for skin permeation of xenobiotics

**DOI:** 10.1038/s41597-020-00764-z

**Published:** 2020-12-01

**Authors:** Dmitri Stepanov, Steven Canipa, Gerhard Wolber

**Affiliations:** 1 Department of Biology, Chemistry & Pharmacy, Institute of Pharmacy, Pharmaceutical and Medicinal Chemistry, Freie Universität Berlin, Berlin, 14195 Germany; 2 Lhasa Limited, Leeds, West Yorkshire LS11 5PS United Kingdom

**Keywords:** Drug delivery, Small molecules

## Abstract

Skin permeation is an essential biological property of small organic compounds our body is exposed to, such as drugs in topic formulations, cosmetics, and environmental toxins. Despite the limited availability of experimental data, there is a lack of systematic analysis and structure. We present a novel resource on skin permeation data that collects all measurements available in the literature and systematically structures experimental conditions. Besides the skin permeation value *k*_*p*_, it includes experimental protocols such as skin source site, skin layer used, preparation technique, storage conditions, as well as test conditions such as temperature, pH as well as the type of donor and acceptor solution. It is important to include these parameters in the assessment of the skin permeation data. In addition, we provide an analysis of physicochemical properties and chemical space coverage, laying the basis for applicability domain determination of insights drawn from the collected data points. The database is freely accessible under https://huskindb.drug-design.de or 10.7303/syn21998881.

## Abstract (summary)

**Table:** 

Measurement(s)	skin permeation of chemicals • skin permeation
Technology Type(s)	digital curation
Factor Type(s)	chemical compound • skin source site • skin layer • skin preparation technique • temperature • storage duration • pH • acceptor medium • donor medium
Sample Characteristic - Organism	Homo sapiens

Machine-accessible metadata file describing the reported data: 10.6084/m9.figshare.13172831

## Background & Summary

Skin is the biggest organ in the human body. It provides a physical barrier to external influences, contributes to the metabolising and excretory functions and can absorb chemical substances. Skin has a layered structure, and it is assumed that a chemical compound, which permeates all layers of epidermis, the uppermost layer of the skin, is being fully carried away into the bloodstream by the capillaries of the dermis^1^. The transport itself is driven by a concentration gradient, and the classical model of permeation describes three absorption pathways: intracellular, intercellular and follicular. Investigation of skin permeation properties of chemical compounds is crucial for occupational exposure risk assessment and cosmetic and pharmaceutical development^1^. For this reason, skin permeation by xenobiotics has been investigated for many years^2–9^. Despite the time and cost intensiveness of the necessary experiments to determine skin permeation properties, the amount of data in the public domain has been steadily growing. This enabled research groups to collect enough data to develop predictive mathematical models. Different modelling approaches were implemented using a multitude of algorithms and data handling procedures. For example, Bratt *et al*.^10^, used permeation values from the Flynn dataset^11^ and removed values, that did not fit the overall data, leaving 60 data points. Chen *et al*.^12^ used permeation data on 215 compounds as a target value and did not report any outlier handling. Degim *et al*.^13^ used data on 40 substances and did not describe any outlier handling steps. A publication by Tsakovska *et al*.^14^ gives a comprehensive overview of these and other record collections.

A careful and thorough analysis of all these approaches revealed that most existing datasets such as the ones compiled by Flynn *et al*.^11^ or Wilschut *et al*.^15^ do not contain the experimental conditions under which the skin permeability measurements were obtained. Others, like the once created by Vecchia *et al*.^16^ or Magnusson *et al*.^17^ include experimental parameters. One of the most comprehensive data collections is the EDETOX database^18^ which returns 537 data points when querying *in vitro* measurements done with human skin. Not all of these data points, however, contain the skin permeation value (or steady-state flux and concentration of the chemical which can be used to calculate skin permeation). Apart from that, EDETOX does not contain the information on the skin source: whether the skin was obtained post-mortem from a cadaver or during a cosmetical procedure. No database containing all experimental conditions and skin permeation records exists to date. Additionally, when carefully comparing and analysing published datasets, inconsistencies can be identified, such as different or simply copied values. For example, Patel *et al*. used a *logk*_*p*_ value for *2-ethoxyethanol* of −7.16 (cm/s). This value was also used by Baba *et al*.^19^, Neely *et al*. (OSU-KP)^20^, Chen *et al*.^12^ and Lian *et al*.^21^. In contrast, Brown *et al*.^22^ used a *logk*_*p*_ value of −6.92 (cm/s). For corticosterone Wilschut *et al*.^15^ and Patel *et al*.^23^ both used a *logk*_*p*_ value of −7.78 (cm/s), but Baba *et al*.^19^ and Lian *et al*.^21^ used a value of −7.08 (cm/s), whereas Lian *et al*. included additional values of −7.56 (cm/s) and −6.81 (cm/s). In addition to this, different research groups used different measuring units. Wilschut *et al*. used *k*_*p*_ measured in cm/h, Patel *et al. logk*_*p*_ in cm/h and Lian *et. al*. *logk*_*p*_ in cm/s. It is also important to consider that different layers of epidermis have different properties in regard to skin permeation: the hydrophobic stratum corneum is a barrier for hydrophilic substances. The hydrophilic viable epidermis, which is positioned under the stratum corneum, represents a barrier to hydrophobic substances. Thus, it is important to consider, which layer of the skin was used to obtain experimental data when analysing skin permeation values.

In this study, we present a novel comprehensive database of skin permeation values and corresponding experimental protocols. This manually curated database is a result of an extensive and careful analysis of previously published human skin permeation datasets and publications containing skin permeation data available in the public domain which additionally includes associated experimental protocols wherever available.

## Methods

We performed a rigorous search and analysis of the original publications that reported skin permeability values to create a comprehensive, systematic and structured database.

### Data content and organisation

Inclusion criteria for the data from a publication were as follows:

- publication was accessible in the public domain
- publication was the primary source of the data
- the *k*_*p*_ value was reported in the paper or it was possible to calculate it from the given steady-state flux and concentration by division (for the sake of data uniformity, huskinDB contains logarithmic *k*_*p*_ values measured in cm/s)
- data was discarded if no quantitative *k*_*p*_ value was reported and it was obtainable only from the plots in the publication
- permeation was measured using undamaged human skin and aqueous solution (in some cases with the addition of ethanol or methanol or in organic solvent) or neat compound.

If the publication satisfied the criteria mentioned above, the following experimental parameters were taken from it in addition to the *k*_*p*_ value:

- name of the compound
- whether the skin was obtained during a surgical procedure or from a cadaver
- site of the body from which skin was excised
- skin preparation technique
- layer of the skin
- storage temperature
- storage duration
- whether the compound was tested neat or as a solution
- temperature of the donor solution
- pH of the donor solution
- type of the donor solution
- temperature of the acceptor solution
- pH of the acceptor solution
- type of the acceptor solution
- type of the permeation cell
- steady-state flux and concentration of the compound (if used for skin permeation calculation)

If parameters were unknown, the corresponding fields were left empty. Apart from the compound name, huskinDB contains canonical SMILES that were generated using Open Babel version 2.4.1 (http://openbabel.org)^24^.

For the purpose of data analysis, molecular weights and octanol/water partition coefficients (logP) were calculated using the RDKit version 2019.09.3 (https://github.com/rdkit/rdkit) library for Python (http://www.python.org).

## Data Records

**Fig. 1:**
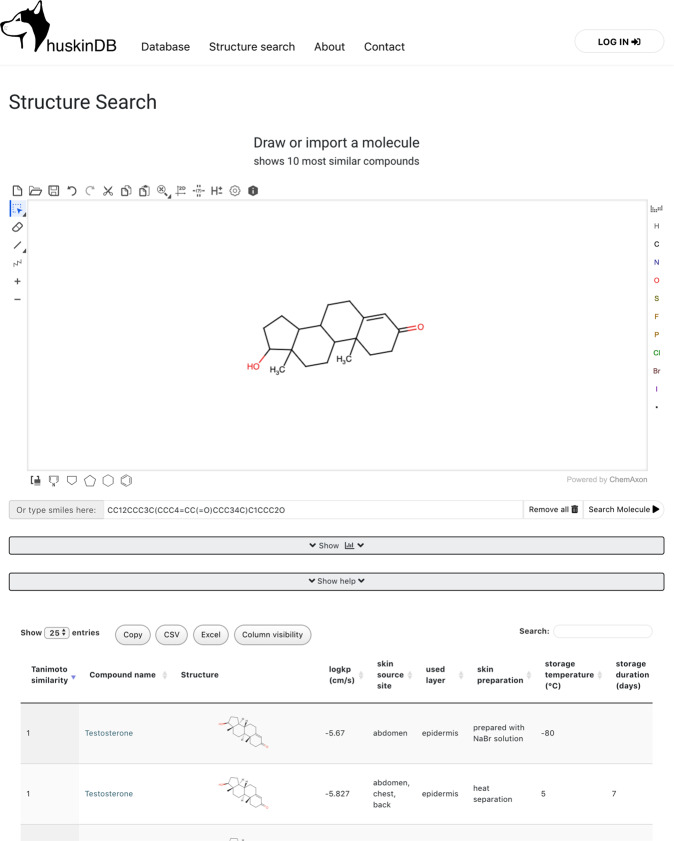
The search section on the huskinDB website allows users to draw a custom molecule and search for similar structures in the database. The user is also able to download the data from any table in the form of either a CSV or Excel file.

Conducted work has led to the development of the “Human Skin DataBase- huskinDB” (huskinDB^25^, 10.7303/syn21998881). As of April 2020, huskinDB^25^ contains 546 *k*_*p*_ values for 251 different compounds covering 94 publications. This data can be accessed also via huskinDB homepage (https://huskindb.drug-design.de). On the huskinDB website, the user has full access to the records in the “Database” section and can perform searches. It is possible to define a custom set of rules to filter the data. If a specific compound is selected, all data on this compound is shown on a separate page. In the “Search” section of the huskinDB website, it is possible to draw a custom molecule using Marvin JS (https://chemaxon.com/products/marvin-js) and search for ten best matching compounds in the database (see Fig. 1). The content of the tables can be downloaded in the form of a CSV-file. Online-only Table 1 gives an overview of the experimental parameters and associated values that describe conditions under which skin permeation values were obtained. These parameters are discussed in greater detail below and correspond to the structure and the content of the downloadable CSV files.

**Table 1:** Thickness of different skin layers can differ depending on the skin source site.

Skin source site	Layer	Thickness (μm)	Reference
All	Stratum corneum	14.8	^45^
	Stratum corneum with Epidermis	83.7	^45^
Abdomen	Epidermis	61.3	^46^
	Epidermis	151	^47^
	Dermis	1844	^47^
	Stratum corneum	6.3	^46^
	Bottom dermal papillae	80.7	^46^
	Epidermis	61.3	^46^
Thigh	Epidermis	55–60	^48^
	Epidermis with dermis	1032–1220	^48^
Back	Stratum corneum	8.4	^46^
	Bottom dermal papillae	75.1	^46^
	Epidermis	55.6	^46^
Breast	Epidermis	419	^49^

Combining the information on the skin source site and thickness can help to assess which layers were used in experiments.

### Compound name

The database contains one name per compound. Only one name was chosen if a different name for the same compound was used in different publications. As shown in Online-only Table 1, the most frequently tested compound was *testosterone* with 16 data points. The second most frequent were *estradiol* and *water* with 15 data points each.

### Skin donor type

Almost half of the skin permeation measurements were assessed using cadaver skin. The rest of the skin was obtained as a result of surgical procedures. The records also specify the type of surgical procedure. If the publication describes using skin from different sources, all of them are given. For example, Anderson *et al*.^26^ describe using skin obtained from a cadaver or during abdominoplasty. Therefore, the corresponding record is “Abdominoplasty or cadaver”.

### Body site (skin source)

Excised abdominal skin was used most frequently, obtained either as a result of abdominoplasty, e.g. Legoabe *et al*.^27^, or removed from a cadaver, e.g. Blank *et al*.^28^. Different sites are listed if different sources were reported in the publication, e.g. Johnson *et al*.^29^ used skin that was removed from the chest, back and abdomen.

### Skin preparation technique

Heat separation was the most frequent method used to prepare the skin. With this technique, previously obtained skin tissue is heated in water at 60°C for one minute, which leads to a separation of the epidermis^30^. The second most frequently reported technique was cutting the skin in different thicknesses using a dermatome - an instrument that cuts the upper layer of the skin of defined thickness^31^. If the thickness of the dermatomed skin was reported, it was noted in the database. If the authors used dermatomed skin but did not report the obtained thickness, no thickness is given, for example, Kushla *et al*.^32^ used dermatomed skin of thickness 150μm and the corresponding entry “dermatomed 150μm” can be found in the in huskinDB, Boogaard *et al*.^33^ did not specify the thickness of dermatomed skin and as such the corresponding entry reads “dermatomed”.

### Layer of the skin used

Analysis of the original publications revealed that research groups used different skin layers to obtain permeability values. As described in the introduction, skin layers have different properties with respect to their absorption of chemicals. This has been recognised by many researchers^26,34–36^. Therefore, it was important for us to include this parameter in the database. If the layer was reported in the original publication, it was directly transferred to huskinDB. Otherwise, the type of the layer was determined from the skin preparation technique, and the body site as follows: if heat separation was used, the corresponding entry is “epidermis”^30^, if the authors wrote that the skin was “cut and placed”, “fat removed” or “dermatomed” without specifying the dermatome thickness, the corresponding entry is “epidermis, dermis”. Otherwise, information from Table 1 was used if the authors reported the thickness of the dermatomed skin and the skin source site. Analysis of the collected data shows that the most frequently used layer was epidermis, followed by epidermis with dermis. This can be explained through the use of heat separation (most frequently used preparation technique) and dermatome (second most frequently used preparation technique) respectively.

The Flux and Notes columns give optional additional information. The first contains steady-state flux and concentration values which were used to calculate skin permeation if it was not directly reported in the publication (by division of the flux value by the concentration). The second one gives clarification on data point obtainment or processing if it was deviating from the overall data point obtainment protocol, e.g. if the *k*_*p*_ measurements was obtained using a suspension as a donor medium.

Other parameters were directly transferred from the original publications.

huskinDB^25^ database is licensed under a Creative Commons Attribution 4.0 International License.

## Technical Validation

The following steps were performed in order to confirm that the records in huskinDB are correctly transferred from the original publications: first, the collected skin permeation values were compared to the ones reported in other data sets if these data sets contained skin permeation values from the same publication. After that, the correctness and completeness of all records was controlled by double-checking the content of the publications which reported experimental data. On this stage of validation errors in approximately 4% of records were identified and corrected. Next, after the initial compilation of huskinDB, all data records were double-checked twice by two different scientists. This validation did not reveal any errors in the data. Subsequently, a random collection of data points (approximately 10%) has been chosen and double-checked once again. No errors were identified on this stage as well.

## Usage Notes

**Fig. 2:**
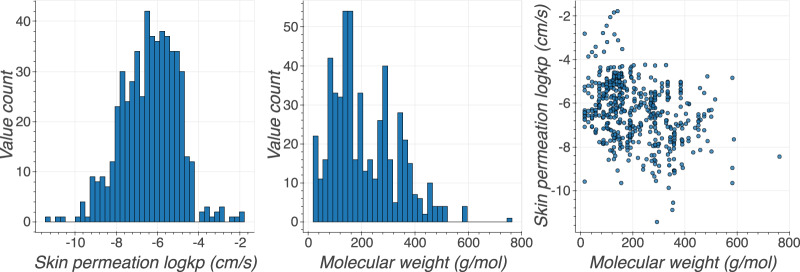
Distribution of all *logk*_*p*_ values (**a**), distribution of molecular weight values (**b**) and plot of molecular weights against *logk*_*p*_ values (**c**) show that huskinDB does not represent all types of molecules equally well.

One of the aims of creating the huskinDB was to facilitate the development of *in silico* methods for predicting skin permeation or to gain additional knowledge of processes behind skin permeation. If developed, such predictive models or insights need to specify their applicability domain, which describes the chemical space in which valid predictions can be performed or to which some hypothesis is applicable^37–39^. This applicability domain directly depends on the database that is used to create predictive models. During an analysis of the previously created models for skin permeation, molecular weight and octanol/water partition coefficient were identified as typical descriptor variables^13,23,40–42^. These two descriptors were also found to improve the accuracy of *logk*_*p*_ predicting models that were developed in-house. Therefore, molecular weight and octanol/water partition coefficient were chosen to analyse the applicability domain of huskinDB. Additionally, skin permeation value (*logk*_*p*_) was used to evaluate the chemical space since applicability domain is bound to its target variable. Below, a brief description of the chemical space of a hypothetical model or hypothesis, that is acquired using the full huskinDB dataset is provided. Obtained skin permeation values range between −11.436 (cm/s) (Propranolol hydrochloride) and −1.778 (cm/s) (1-Nonalol) with a mean value of −6.36 (cm/s) (SD = 1.36, SE = 0.059). This is illustrated in Fig. 2(a), where *logk*_*p*_ values in the histogram form a skewed Gaussian distribution. Digitoxin (764.4) and disodium octaborate tetrahydrate (590.0) show the largest molecular weight, whilst water (18.0) and methanol (32.0) form the lower molecular weight limit. A histogram of molecular weights in Fig. 2(b) shows a bias towards compounds with lower values. A plot of molecular weight against *logk*_*p*_ in Fig. 2(c) reveals that the coverage of the chemical space is non-homogeneous.

**Fig. 3:**
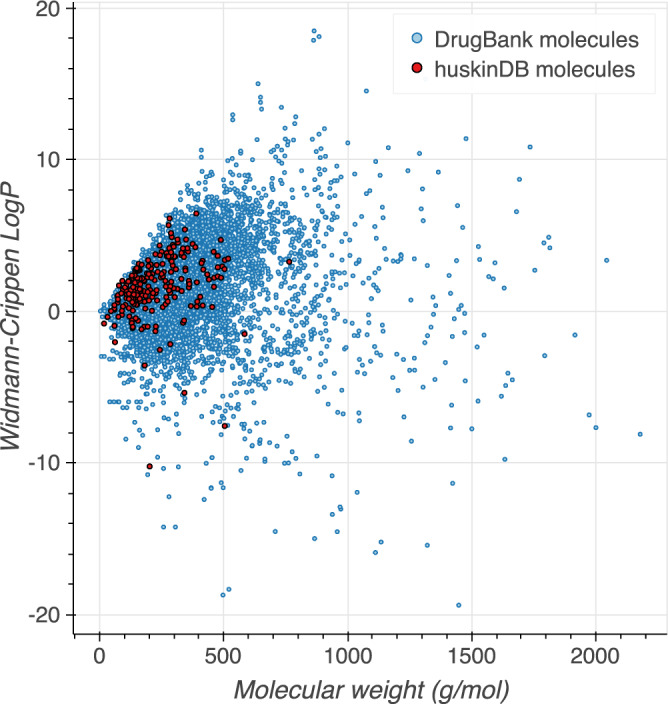
Comparison of DrugBank and huskinDB data over molecular weight and calculated logP (Wildman-Crippen logP) shows that a hypothetical model that is based on all huskinDB compounds, will not give equally reliable predictions for all compounds in DrugBank.

For chemical space analysis, 8,697 molecules were obtained from the DrugBank version 5.0.11^43^ (37 molecules were omitted: it was either not possible to process them with RDKit, or their molecular weight was larger than 2,500 or their calculated *logP* was higher than 20 or less than −20). The distribution of data from DrugBank and huskinDB over molecular weight and octanol/water partition coefficient (calculated with RDKit) in Fig. 3 illustrates that compounds from huskinDB only represent a subset of the DrugBank molecules: they show a smaller spread on both axes and represent molecules with relatively small weights.

Additional information on experimental conditions in huskinDB can be used to explain phenomena that were not correctly interpreted or understood before. In the following example, this use case is shown on the skin permeation values of three steroid subsets that were found by research groups to be inconsistent with other measurements: 1995, Barratt *et al*.^10^ identified 12 measurements of hydrocortisone derivatives in the Flynn dataset that were described as outliers. All of these measurements were performed by the same research group - Anderson *et al*.^26^ (further referred to as Anderson steroids). Later, Abraham *et al*.^44^ reported that the permeation values of steroids measured by Scheuplein *et al*.^5^ (Scheuplein steroids) are too low in comparison to more recent values published by Johnson *et al*.^29^ (Johnson steroids). To assess the correctness of both steroid data subsets, Abraham *et al*.^44^ developed a model that was not based on any of these values and evaluated its predictions. Abraham *et al*.^44^ showed that predictions are in better agreement with Johnson’s data. Another interesting observation was made by Abraham *et al*., who suggested that Anderson steroids have *k*_*p*_ values that are too high.

**Fig. 4:**
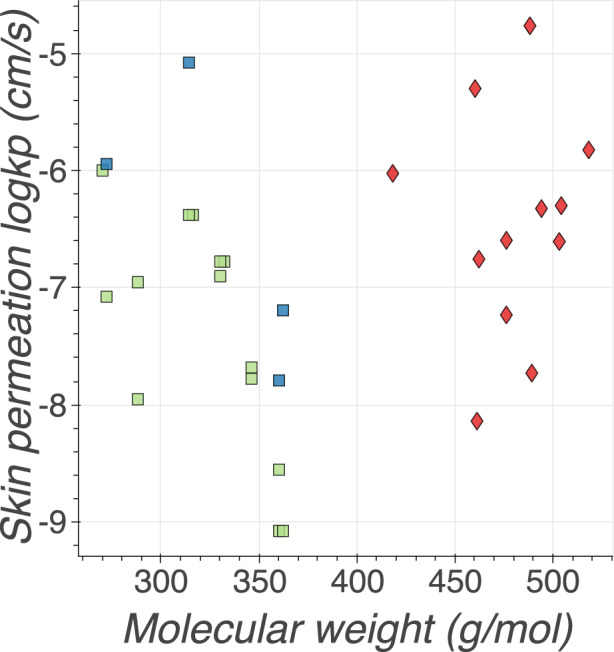
Steroids measured by Scheuplein, Johnson and Anderson are grouped in clusters. It is apparent that these researchers used compounds with different molecular weight classes and performed experiments with different skin layers; in green: Scheuplein steroids, in blue: Johnson steroids, in red: Anderson steroids; the layer of the skin that was used is shown by the shape of the marker – square: epidermis, diamond: stratum corneum. Scheuplein steroids, in blue: Johnson steroids, in red: Anderson steroids; the layer of the skin that was used is shown by the shape of the marker – square: epidermis, diamond: stratum corneum.

Figure 4 shows the relationship between *logk*_*p*_ values and the molecular weight of three steroid subsets. The permeation values of different groups are clustered; however, when analysed more closely, it is apparent that the data points are grouped based on the molecular weights. Further data analysis reveals that these values were obtained using different skin layers. Permeation of Scheuplein and Johnson steroids were measured using epidermis, whereas Anderson steroids were measured using thinner layer- stratum corneum. This means that the difference in the skin permeability between the steroid subsets might be caused by the difference in molecular weights on the one hand and by different skin layers on the other.

This demonstrates that the application area of huskinDB goes beyond permeation value prediction. It can also be used to assess the influence of experimental variables on the *k*_*p*_ value. However, it must be noted that huskinDB contains molecules with small molecular weights in comparison to databases such as DrugBank^43^. Apart from that, data on experimental protocols of many measurements is unknown. Therefore, a direct comparison of the data points is not always possible. For example, misleading conclusions can be drawn when comparing two measurements with one obtained using aqueous solution and another using unknown donor medium, which may be non-aqueous. Careful analysis of the skin membrane with respect to the lipophilicity of the chemical compound is advised as well when looking at a specific skin permeation measurement. A skin membrane consisting solely of stratum corneum will provide a weaker barrier function than a membrane consisting of the whole epidermis and dermis, especially for the lipophilic compounds since the hydrophilic viable epidermis may become the rate-determining factor.

Also, we would like to point out that the user might not be interested in the *k*_*p*_ value itself, rather in the flux values across the skin. These values might provide a descriptor more adequate for estimation of the compound intake during, for example, occupational exposure and can be calculated by multiplication of the *k*_*p*_ value by the compound concentration in the donor medium. Given that the compound solubility is used as the concentration, one might calculate the theoretical maximum flux. The solubility, in its turn, is dependent on the medium in which the compound is dissolved. This also denotes the importance of the data point quality validation since the donor medium and thus the solubility is different across the data records in huskinDB, making direct *k*_*p*_ value comparison rather challenging and, in some cases, even misleading. Different properties of different solvents also mean that the *k*_*p*_ value of some compound obtained using one solvent, cannot be used to predict the flux value of the same compound in another solvent. Therefore, it is vital for the user to perform a proper data handling and to account for the limited chemical space and unknown values prior creating mathematical models or drawing conclusions from the data. In this regard, defining a custom set of rules would rank the data point suitability for one’s domain of interest in a tailored way. For example, a measurement obtained using a donor solution containing 90% ethanol or a measurement with many unknown parameters might be ranked as a “low confidence record” and excluded from further processing. In contrast, a data point obtained using common and known parameters such as phosphate-buffered saline as donor and acceptor medium at 37 °C and heat-separated epidermis would be ranked as a “high confidence record” and included in subsequent analysis. It is also important to note that more reliable data on compounds from different chemical classes would further expand the applicability domain of huskinDB. We plan to update the database, and therefore researchers are encouraged to submit their data to huskinDB, which provides a technical framework for the structured deposition of skin permeability data for small organic molecules.

## Data Availability

The code which was used to create Figs. 2–4 and analyse the data records in huskinDB can be found under the following link: https://github.com/RhDm/huskinDB_publication This repository contains a detailed guide on how to install the requirements and run the code.

## Online-only Table

**Online-only Table 1:** As of April 2020, huskinDB contains 546 permeability values of 251 unique compounds and corresponding experimental parameters.

Experimental parameter	Entry	Number of entries	Entry	Number of the entries
Compound name (top 10)	Testosterone	16	1-Hexanol	13
	Estradiol	15	N,N-Diethyl-m-toluamide	12
	Water	15	Fentanyl	12
	1-Butanol	14	Ethanol	11
	1-Octanol	14	Sufentanil	11
			Total (known):	546
			Unique values:	251
Skin donor type	Cadaver	260	Abdominoplasty or mammaplasty	17
	Abdominoplasty	110	Abdominoplasty or cadaver	16
	Unknown	67	Amputated limb	11
	Mammaplasty	34	Mammaplasty or cadaver	2
	Surgery	29		
			Total (known):	546
			Unique values:	9
Body site (skin source) (top 10)	Abdomen	302	Abdomen, chest, back	9
	Unknown	88	Abdomen, back	7
	Thigh	60	Torso	6
	Breast	35	Leg	5
	Abdomen, breast	17	Scalp	4
			Total (known):	458
			Unique values:	16
Skin preparation (top 10)	Heat separation	217	Exposure to ammonia fumes	19
	Unknown	107	Dermatomed 250–280μm	18
	Fat removed	31	Prepared with NaBr solution	16
	Dermatomed	23	Dermatomed 500μm	16
	Dry heat separation	22	Heat separation, hydrated	10
			Total (known):	439
			Unique values:	30
Layer of the skin used	Epidermis	230	Dermis	33
	Epidermis, dermis	228	Unknown	14
	Stratum corneum	35	Epidermis without stratum corneum	4
			Total (known):	532
			Unique values	7
Storage temperature (°C) (top 10)	unknown	162	-30	11
	4	140	−25	10
	−20	139	5	9
	−70	20	−17	7
	−80	20	−26	6
			Total (known):	384
			Unique values:	16
Storage duration (days)	Unknown	385	120	10
(top 10)	14	26	28	9
	7	19	60	9
	8.5	16	180	8
	90	13	0.5	8
			Total (known):	161
			Unique values:	18
Compound applied as	Solution	497	Neat	49
			Total (known):	546
			Unique values:	2
Donor temperature (°C)	Unknown	155	35	18
	37	114	26	14
	32	90	31	10
	25	80	40	8
	30	53	22	4
			Total (known):	391
			Unique values:	10
Donor pH (top 10)	Unknown	322	7.5	5
	7.4	83	7.3	4
	7	31	5.0	4
	4	23	2	3
	9.7	6	10	3
			Total (known):	224
			Unique values:	54
Donor type (top 10)	Unknown	238	NaCl, sodium azide solution	15
	PBS	90	Citrate-phosphate buffer	14
	Water	46	Potassium phosphate buffer	12
	Succinate solution	22	Sorensen’s buffer	8
	NaCl solution	17	Propylene glycol/water (1:1)	7
			Total (known):	308
			Unique values:	48
Acceptor temperature	37	194	35	18
(°C) (top 10)	Unknown	152	26	14
	32	64	31	9
	30	46	36	4
	25	39	22	4
			Total (known):	394
			Unique values:	11
Acceptor pH (top 10)	Unknown	298	7	10
	7.4	171	7.1	10
	6.5	17	7.6	4
	4	16	7.5	4
	7.3	11	4.5	2
			Total (known):	248
			Unique values:	13
Acceptor type (top 10)	Unknown	171	Succinate solution	16
	PBS	128	PBS with bovine serum albumin	15
	Water	46	PBS with ethanol	14
	NaCl solution	42	Sodium Azide	11
	Ringer’s solution	23	Dulbecco PBS	8
			Total (known):	375
			Unique values:	32
Cell type	Glass diffusion cell	152	Pyrex cell	64
	Franz cell	147	Static diffusion cell	6
	Unknown	106	Semi-automated apparatus	2
	Flow through cell	68	Teflon cell	1
			Total (known):	440
			Unique values:	8

If the database contains more than 10 different values of some parameter, only the 10 most frequent values are shown (top 10). HEPES: 4-(2-hydroxyethyl)−1-piperazineethanesulfonic acid), HBSS: Hank’s balanced salt solution, PBS: Phosphate buffered saline.
